# Synthesis of Coumarin-Based Photosensitizers for Enhanced Antibacterial Type I/II Photodynamic Therapy

**DOI:** 10.3390/molecules29163793

**Published:** 2024-08-10

**Authors:** Min Ma, Lili Luo, Libing Liu, Yuxuan Ding, Yixuan Dong, Bing Fang

**Affiliations:** 1 Department of Nutrition and Health, China Agricultural University, Beijing 100193, China; mamin_st@163.com (M.M.); lililuo_w@163.com (L.L.); dingyxuan2024@163.com (Y.D.); dongyuxuan322@163.com (Y.D.); 2 Key Laboratory of Precision Nutrition and Food Quality, China Agricultural University, Beijing 100193, China

**Keywords:** type I photodynamic, aggregation-induced emission materials, photosensitizers, fluorescence imaging

## Abstract

Photodynamic therapy (PDT) is an effective method for treating microbial infections by leveraging the unique photophysical properties of photosensitizing agents, but issues such as fluorescence quenching and the restricted generation of reactive oxygen species (ROS) under hypoxic conditions still remain. In this study, we successfully synthesized and designed a coumarin-based aggregation-induced emission luminogen (AIEgen), called ICM, that shows a remarkable capacity for type I ROS and type II ROS generation. The ^1^O_2_ yield of ICM is 0.839. The ROS it produces include hydroxyl radicals (HO^•^) and superoxide anions (O_2_^•−^), with highly effective antibacterial properties specifically targeting *Staphylococcus aureus* (a Gram-positive bacterium). Furthermore, ICM enables broad-spectrum fluorescence imaging and exhibits excellent biocompatibility. Consequently, ICM, as a potent type I photosensitizer for eliminating pathogenic microorganisms, represents a promising tool in addressing the threat posed by these pathogens.

## 1. Introduction

Human infections with pathogenic microorganisms primarily occur when these pathogens invade the human body through direct or indirect contact. The rise of antibiotic and antifungal resistance in these “superbugs” and “superfungi” has become a global concern, causing alarm and even panic [1,2,3]. Therefore, it is imperative to diagnose infectious diseases quickly and accurately, in close combination with clinical practice to put forward reliable and valid treatment plans that can prevent drug resistance and nosocomial infections [4]. In contrast to the hidden dangers of drug resistance and the heavy toxic side effects associated with traditional therapies, photodynamic therapy (PDT) has shown promise in the treatment of pathogenic microorganisms due to its crucial advantages of a lower susceptibility to drug resistance, spatial and temporal controllability, and non-invasiveness [5,6,7,8]. Currently, critical progress has been made in the preparation of photosensitizers. However, traditional photosensitizers are more prone to showing an aggregation-caused quenching (ACQ) effect in molecular species, which results in a low fluorescence intensity and low generation of reactive oxygen species (ROS) [9]. Compounds displaying aggregation-induced emission (AIE) demonstrate fascinating properties due to their unique molecular structure in an aggregated state, giving them the potential to overcome the ACQ effect. An AIE luminogen (AIEgen) is unable to effectively emit light in its monomeric state [10,11]. However, when an AIEgen is aggregated or embedded in a solid matrix, its motions are restricted, leading to the dissipation of excited-state energy mainly through radiative pathways, resulting in intense fluorescence [12,13,14]. Therefore, AIEgens are excellent candidate photosensitizers suitable for PDT.

**Scheme 1:**
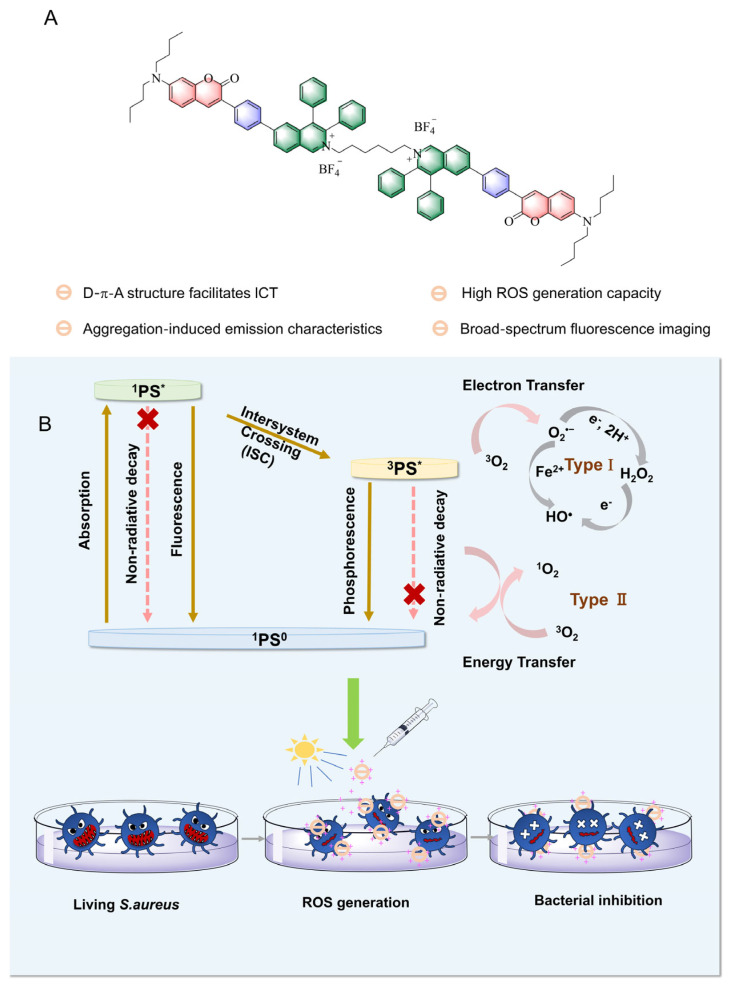
(**A**) Molecular structures of ICM. (**B**) Illustration of photophysical mechanisms of Type I and Type II photosensitizers and antibacterial application of ICM.

Upon illumination, a photosensitizer transitions from the ground state (S_0_) to the transient excited state (S_1_) and undergoes intersystem crossing (ISC) to the stable triplet state (T_1_). Photosensitizers in T_1_ can generate ROS through two distinct pathways [15]. Currently, the majority of PDT involves type II mechanisms, which rely on oxygen to generate singlet oxygen (^1^O_2_) [16]. As a result, it is difficult to achieve a bactericidal effect on pathogenic microorganisms in oxygen-deficient environments. Therefore, the development of type I photosensitizers that can efficiently generate ROS in hypoxic conditions is of great importance. Type I PDT produces ROS such as hydroxyl radicals (HO^•^) and superoxide anions (O_2_^•−^) through electron transfer [17,18,19]. Although type I PDT is more suitable for use in hypoxic environments, it still requires the participation of oxygen and cannot react independently of oxygen [20]. Type II PDT directly transfers energy to oxygen molecules to produce highly active ^1^O_2_ (Scheme 1B). Oxygen plays a vital role in the whole reaction process [21]. Generally, these two mechanisms in PDT compete with each other, with the type II mechanism occurring faster than the type I mechanism [22,23]. Therefore, via feasible structural adjustment of the photosensitizer, it is possible to enhance the photosensitization effect and achieve efficient concomitant production of ROS, thereby realizing precise and effective PDT. A common method to enhance the photosensitization efficiency of a photosensitizer is to increase the ISC efficiency between molecules [24]. According to perturbation theory, increasing the rate constant (*k*_ISC_) of ISC can elevate the triplet excitons and, consequently, increase the ISC efficiency. Augmenting *k*_ISC_ can reduce the energy gap (ΔE_ST_) between S1 and T1 by enhancing intramolecular charge transfer (ICT) [25]. Introducing a donor–π–acceptor (D-π-A) structure to modulate the π-conjugation in organic conjugated molecules adjusts the distribution of electron clouds between the highest occupied molecular orbital (HOMO) and the lowest unoccupied molecular orbital (LUMO), resulting in increased electron delocalization and decreased electronic repulsion, effectively reducing ΔE_ST_ and enhancing ISC efficiency [26,27,28]. Based on the aforementioned design principles, feasible structural adjustments and molecular modifications to photosensitizers can promote their targeting and corresponding functions, thus realizing efficient concomitant production of ROS for PDT.

Coumarin possesses pharmacological properties such as anticoagulant, antibacterial, anti-inflammatory, and anticancer effects [29,30,31]. It also exhibits excellent luminescent properties due to its inherent charge transfer characteristics. Furthermore, the heterocyclic compounds with a coumarin core show favorable photophysical and pharmacokinetic properties when the heterocycles are introduced into the coumarin unit. π-extended coumarin derivatives with strong electron-donating abilities and the presence of oxygen heteroatoms exhibit outstanding photophysical and photochemical properties as well as good biocompatibility [32]. They also exhibit stronger affinity for pathogenic microorganisms, showing potential in the treatment of microbial infections.

In this study, we propose an AIEgen called ICM, with a binary (D-π-A)–linker–(D-π-A) structure, in which flexible alkyl chains serve as the connecting units. Introducing a rotor-type diphenyl isoquinolinium as the receptor ensures the AIE properties, while incorporating a pyridinium salt structure with a positive charge enhances the molecule’s hydrophilicity, effectively promoting the binding with negatively charged pathogenic microorganisms via electrostatic interactions, thereby resulting in an AIEgen with intramolecular charge transfer (ICT) effects. ICM demonstrates a paramount ability to produce ROS, particularly HO^•^ and ^1^O_2_. Under irradiation with a low white-light intensity (5mW/cm^2^) for 15 min, 2 µM ICM achieved a killing rate of 96% against *Staphylococcus aureus* (*S. aureus*). Furthermore, ICM provided broad-spectrum fluorescent imaging of *S. aureus* (a Gram-positive bacterium), *Escherichia coli* (*E. coli*, a Gram-negative bacterium), and *Candida albicans* (*C. albicans*, a fungus). Detailed studies revealed that ICM has strong type I ROS and type II ROS generation capability with high efficiency in killing *S. aureus*.

## 2. Results and Discussion

### 2.1. Synthesis and Optical Performance of ICM

**Scheme 2:**
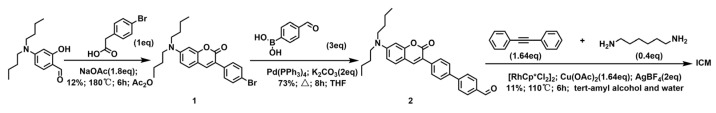
The synthetic route of ICM.

The chemical structure of ICM is shown in Scheme 1A. In order to obtain an efficient photosensitizer with high ROS generation capability, we designed a (D-π-A)–linker–(D-π-A)-type cationic photosensitizer connected by a flexible alkyl chain. ICM consists of a receptor unit (isoquinolinium unit), a π-bridge unit (phenyl ring), and a donor unit (coumarin derivative). Compound 1 (yield: 12%) was obtained via the condensation of salicylaldehyde derivative and 4-bromophenylacetic acid using Ac_2_O, followed by a Suzuki coupling reaction with (4-formylphenyl)boronic acid catalyzed by Pd(PPh_3_)_4_ to afford compound 3 (yield: 73%). Finally, ICM was synthesized using a one-pot synthesis method (yield: 11%). The synthetic route of ICM is shown in Scheme 2, and the chemical structure was characterized by means of ^1^H NMR, ^13^C NMR, and HRMS (Figures S1–S7).

**Figure 1:**
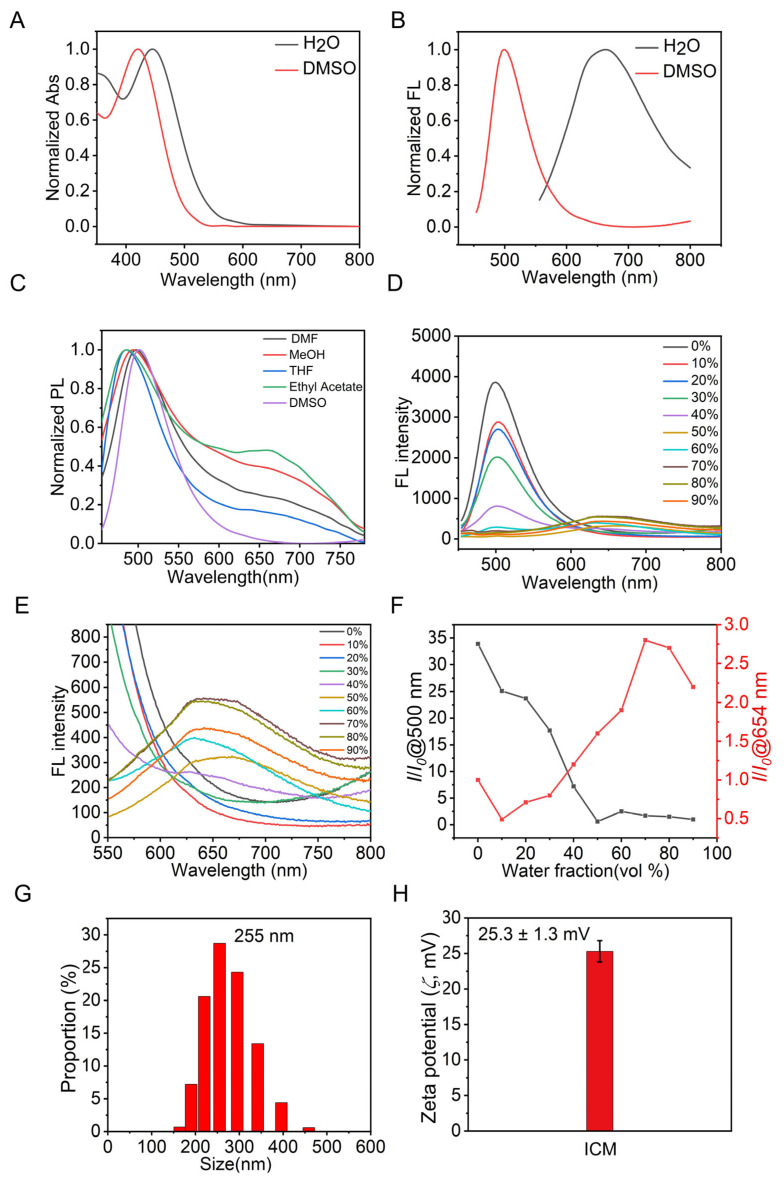
(**A**) Normalized UV–vis spectra of 10 μM ICM in H_2_O/DMSO; (**B**) normalized fluorescence spectra of 10 μM ICM in H_2_O/DMSO; (**C**) normalized fluorescence spectra in different solvents (excitation at 430 nm); (**D**) fluorescence spectra of ICM in DMSO solvent with different water fractions; (**E**) amplified fluorescence spectra at 654 nm; (**F**) line chart of relative fluorescence intensity (I/I_0_) at 500 nm and 654 nm, with I_0_ at 90% or 0% water fractions; (**G**) zeta potential of ICM; (**H**) DLS data of ICM.

The photophysical properties of ICM in H_2_O and DMSO solvents were studied through UV/visible absorption spectroscopy and fluorescence emission spectroscopy (Figure 1A,B). The maximum absorptions of ICM were located at 418 nm in DMSO and 447 nm in H_2_O (Figure 1); ICM’s absorption spectroscopy was essentially unaffected by different solvents. (Figure S8). The maximum fluorescence emission wavelengths in H_2_O and DMSO were around 600 to 660 nm and 450 to 550 nm, respectively, indicating that solvents of different polarities have a certain effect on the emission spectral shift. Furthermore, the spectra demonstrated large Stokes shifts, which are advantageous for avoiding low signal-to-noise ratios and severe fluorescence self-quenching phenomena when applied in biological imaging [33]. The emission spectra of ICM in different solvents revealed a continuous redshift of the emission wavelength with changing solvent polarity due to its twisted D-*π*-A structure [34]. In highly polar water, the emission peak was located near 654 nm (Figure 1C). AIE properties are typically verified using good solvent/poor solvent systems. With the addition of a poor solvent to a good solvent, AIEgens aggregate, leading to a redshift in wavelength and an increase in fluorescence intensity. ICM exhibits typical AIE characteristics. As the water volume increased in the DMSO solution of ICM, the wavelength underwent a redshift due to the typical twisted intramolecular charge transfer (TICT) effect [35] (Figure 1D). The fluorescence intensity at 500 nm decreased, and after the water content exceeded 50%, the fluorescence intensity at 654 nm increased (Figure 1E,F). The fluorescence quantum yields of ICM reached 0.102% in the aggregation state, which was about 1.44-fold of those in the DMSO solution (0.071%) (Table S1). Further evidence of ICM forming aggregates through self-assembly was obtained via dynamic light scattering (DLS) measurements, showing a zeta potential of 25.3 ± 1.3 mV and an average diameter of 255 nm (Figure 1G,H).

### 2.2. ROS Generation Evaluation and Theoretical Calculation

**Figure 2:**
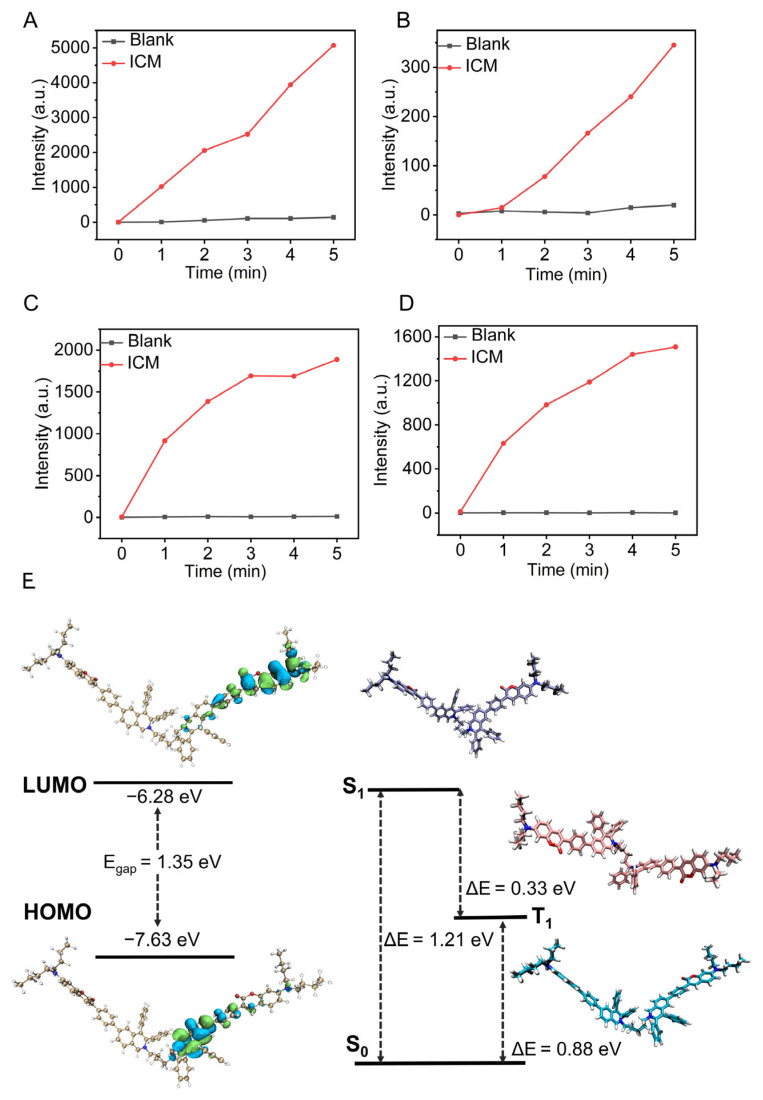
(**A**) Fluorescence chart for the detection of total ROS with 50 nM ICM and DCFH under 5 mW/cm^2^ white light irradiation, with measurements taken every minute; (**B**) detection of ^1^O_2_ generation with enhancement of SOSG for 0.5 μM ICM under 5 mW/cm^2^ light irradiation; (**C**) detection of O_2_^•−^ generation with the decomposition of DHR123 for 0.5 μM ICM under 5 mW/cm^2^ light irradiation; (**D**) detection of HO^•^ with 0.5 μM ICM and HPF upon 5 mW/cm^2^ white light irradiation; (**E**) calculated frontier molecular orbitals, with the ΔE_S1T1_ value for ICM used in the Gaussian 09 program package at the B3LPY/6-31G (d, p) level.

We utilized the commercial probes 2′,7′-dichlorodihydrofluorescein diacetate (DCFH-DA), the Singlet Oxygen Sensor Green (SOSG) fluorescent probe, hydroxyphenyl fluorescein (HPF), and dihydrorhodamine 123 (DHR123) to measure the generation capacities for total ROS, ^1^O_2_, HO^•^, and O_2_^•−^, respectively. As depicted in Figure 2A, DCFH can be oxidized by ROS to form 2′,7′-dichlorofluorescein (DCF), and the change in the fluorescence intensity of DCF is positively correlated with the ROS generation capacity. In the presence of photosensitizers, under 5 mW/cm^2^ white light irradiation, the fluorescence intensity of the DCFH increased with prolonged irradiation time. After continuous irradiation for 5 min, the fluorescence intensity of the control group containing only the DCFH solution was negligible. In further investigations of other types of ROS generation, HPF exhibited a rapid increase in fluorescence intensity after interaction with 0.5 μM ICM (Figure 2D), demonstrating a significant HO^•^ generation capacity of ICM. When DHR123 solution was treated with 0.5 μM ICM and exposed to 5 mW/cm^2^ white light for 5 min, the fluorescence intensity increased accordingly (Figure 2C), fully illustrating the ability of ICM to generate O_2_^•−^ under light irradiation. Furthermore, when SOSG was used to validate the generation of ^1^O_2_, the fluorescence intensity increased after interaction with ICM (Figure 2B), indicating that ICM also possesses a certain capacity for ^1^O_2_ generation and that the occurrence of the type I mechanism did not completely inhibit the type II mechanism, further suggesting that ICM can simultaneously generate type I and type II reactive oxygen species. Furthermore, the ^1^O_2_ quantum yield of ICM was measured to be 0.839 using 9,10-anthracenediyl-bis-(methylene)dimalonic acid (ABDA) as an indicator and rose bengal (RB) as a standard reference (Figure S9) [36,37].

In order to delve into the mechanism of ROS generation in type I/II PDT, as illustrated in Figure 2E, the B3LPY/6-31G (d, p) method was employed to optimize the structures in the ground state and excited states (S1, T1). The ISC process (S1–T1) of ICM was found to have an energy gap (ΔE_S1T1_) of 0.33 eV. The distribution of electron clouds in the HOMO and LUMO of ICM leads to HOMO–LUMO separation, facilitating the ISC process and favoring ROS generation [38]. The HOMO–LUMO energy gap for this ICM was determined to be 1.25 eV. The HOMO electron cloud was mainly distributed near the coumarin derivative and benzene ring, while the electron clouds of the LUMO orbital were primarily located in the diphenylisoquinoline part, indicating the separation of the HOMO–LUMO orbitals and typical D-π-A characteristics.

### 2.3. Pathogenic Microorganism Imaging and Photodynamic Antimicrobial Study

**Figure 3:**
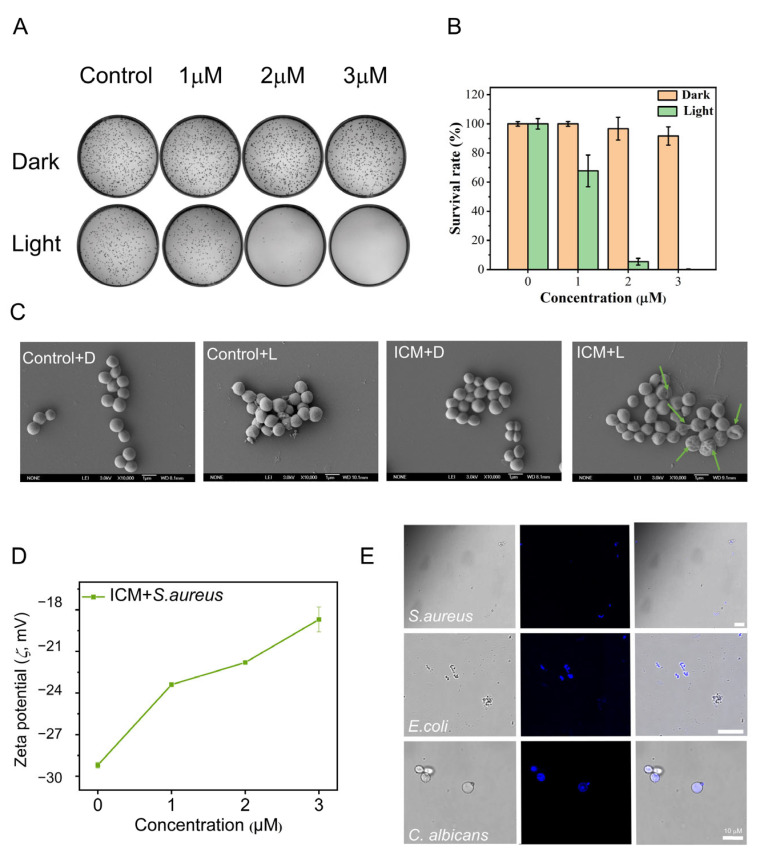
(**A**) *S. aureus* and different concentrations of ICM on agar plates under light (5 mW/cm^2^) and dark conditions; (**B**) survival rate graph of *S. aureus* treated with different concentrations of ICM; (**C**) SEM images of *S. aureus* incubated with various concentrations of ICM (scale bar: 1 μm); (**D**) zeta potential of *S. aureus* treated with different concentrations of ICM; (**E**) CLSM images of ICM interacting with different pathogenic microorganisms (scale bar: 10 μm).

Based on the aforementioned results, the bactericidal performance of ICM against *S. aureus* (Gram-positive bacteria), *E. coli* (Gram-negative bacteria), and *C. albicans* (fungi) was evaluated using a plate counting method. As shown in Figure 3A,B, untreated *S. aureus* showed no changes under dark or light conditions. Under exposure to a very low light intensity (5 mW/cm^2^), *S. aureus* exhibited only 5.4% survival efficiency against 2 µM ICM, while 3 µM ICM eradicated all *S. aureus*. ICM’s excellent antibacterial performance against *S. aureus* can possibly be attributed to the alkalinity provided by the presence of butylamino, which enhances its binding with the teichoic acid on the surface of *S. aureus* cells [39]. ICM showed minimal dark toxicity and demonstrated enhanced biocompatibility when combined with PDT. Furthermore, cationic ICM is more prone to binding with pathogens carrying negative surface charges, and there are reports in the literature that the number of positive charges plays a promoting role in a compound’s antimicrobial efficiency; hence, ICM with two positive charges displayed more important antibacterial effects [40]. *E. coli* exhibited a survival efficiency of only 47% against 20 µM ICM (Figure S10B,D). The bactericidal efficiency of ICM against *S. aureus* was stronger compared with that against *E. coli*, possibly due to the presence of an outer membrane in *E. coli* hindering the interaction between the photosensitizer and the bacteria [41]. *C. albicans* displayed a survival efficiency of 8.3% against 0.1 mM ICM (Figure S10A,C). Thus, ICM exhibited specific PDT killing effects, at low concentrations, against *S. aureus*. To further explore the antibacterial mechanisms of ICM, changes in the zeta potential of *S. aureus*, *E. coli*, and *C. albicans* were studied. When incubated together with *S. aureus* for 15 min, ICM caused a positive shift in the potential, indicating effective binding of ICM with *S. aureus* through electrostatic interactions, leading to ICM’s high antibacterial efficiency against *S. aureus* (Figure 3D). It is noteworthy that at a high concentration of 20 µM, *E. coli* showed a slight positive shift in its zeta potential after interaction with ICM (Figure S10F). This suggests that ICM also binds to *E. coli* through electrostatic interactions but the binding capacity is weaker at low concentrations due to the presence of the outer membrane in Gram-negative bacteria. After interaction with ICM, the zeta potential of *C. albicans* exhibited a noticeable positive shift with increasing concentrations of ICM (10 µM, 20 µM, 0.1 mM) (Figure S10E), indicating binding through electrostatic interactions. These zeta potential results are consistent with the results of the photodynamic antibacterial studies, further supporting the hypothesis that ICM may bind to the cell membranes of pathogenic microorganisms through electrostatic interactions, leading to the disruption of microbial cell membranes and subsequent eradication of the pathogens.

A further investigation was carried out on the binding and luminescence of ICM with different pathogenic microorganisms to confirm their strong binding interactions. After incubation with 0.5 µM ICM for 15 min, a bright blue fluorescence was observed in the fluorescence field during interaction with *S. aureus*. Furthermore, ICM demonstrated staining capabilities against *E. coli* and *C. albicans* (Figure 3E). Hence, ICM can bind to *S. aureus*, *E. coli*, and *C. albicans* through electrostatic interactions, enabling broad-spectrum fluorescence imaging.

Subsequently, scanning electron microscopy (SEM) was employed for a visual assessment (The green arrows indicated bacterial cell shrinkage and rupture). In the control group, *S. aureus* exhibited a smooth extracellular edge and a well-rounded morphology under both light and dark conditions. After treatment with 3 μM ICM without light exposure, the bacterial cells maintained a healthy state; however, upon exposure to 5 mW/cm^2^ light, the *S. aureus* cell membrane collapsed, resulting in wrinkling and rupture that released the cell contents, showcasing the strong antibacterial effect of PDT (Figure 3C). After light exposure, upon interaction with 20 μM ICM, most of the *E. coli* cells displayed surface pits and adhesion at their edges. Under light conditions, 0.1 mM ICM exhibited almost complete eradication of *C. albicans* upon interaction (Figure S9G). The SEM images were consistent with the results obtained via the plate counting method, and they confirmed the ability of ICM to achieve microbial eradication by disrupting the cell membrane. The biocompatibility of ICM was further validated through a CCK-8 assay (Figure S11). The compound exhibited negligible cytotoxicity at the experimental concentration, indicating ICM as a biocompatible photosensitizer that poses no harm to biological tissues.

## 3. Materials and Methods

### 3.1. Materials

Chemical reagents were commercially purchased without the need for purification. All chemical reagents were sourced from TCI, Sigma-Aldrich (St. Louis, MO, USA), or Macklin Co., Ltd. (Shanghai, China). Chemical solvents were obtained from Beijing Chemical Reagents Co., Ltd. (Beijing, China). Bacterial culture-related consumables were obtained from Solarbio (Beijing, China), and the bacterial strains were sourced from the China General Microbiological Culture Collection Center.

### 3.2. Synthesis of Compound ***1***

4-(Dibutylamino) salicylaldehyde (2.0 g, 8.02 mmol, 1 eq), 4-bromophenylacetic acid (1.7 g, 8.02 mmol, 1 eq), and anhydrous CH_3_COONa (1.2 g, 14.4 mmol, 1.8 eq) were dissolved in Ac_2_O (10 mL). The reaction mixture was refluxed at 180 °C under a nitrogen atmosphere for 6 h. After the completion of the reaction, the mixture was cooled to room temperature. Water was added to quench the reaction, followed by extraction with chloroform. The organic phase was dried over anhydrous magnesium sulfate to remove residual water, and the crude product was purified using a silica gel column with an eluent of n-hexane/ethyl acetate (*v*/*v* = 6/1) to obtain a final yellow powder (12% yield, 0.4 g). ^1^H-NMR (500 MHz, CDCl_3_): δ 7.68 (s, 1H, coumarin-H), 7.59–7.51 (m, 4H, Ar-H), 7.30 (d, 1H, *J* = 8.5 Hz, coumarin-H), 6.58 (dd, 1H, *J* = 8.5, 2.5 Hz, coumarin-H), 6.49 (d, 1H, *J* = 4.0 Hz, coumarin-H), 3.35 (t, 4H, *J* = 7.5 Hz, -NCH_2_), 1.64–1.57 (m, 4H, -CH_2_), 1.42–1.34 (m, 4H, -CH_2_), 0.99 (t, 6H, *J* = 7.5 Hz, -CH_3_); ^13^C NMR (125 MHz, CDCl_3_), δ 161.44, 156.24, 151.14, 140.60, 134.75, 131.40, 129.79, 128.94, 121.70, 119.31, 109.19, 108.85, 97.17, 51.08, 29.27, 20.26, 13.95.

### 3.3. Synthesis of Compound ***2***

Compound **1** (0.010 g, 0.23 mmol, 1 eq) and 4-formylphenylboronic acid (0.105 g, 0.70 mmol, 3 eq) were dissolved in 10 mL of THF. The reaction mixture was heated and stirred at 50 °C under a nitrogen atmosphere, and then 5 mg Pd(PPh_3_)_4_ was added. After 15 min, a 1 M K_2_CO_3_ solution (460 μL, 2 eq) was added, and the reaction mixture was refluxed at 80 °C for 8 h. After the completion of the reaction, the mixture was quenched with water and extracted with dichloromethane. The organic phase was combined and dried over anhydrous magnesium sulfate. The solvent was removed via rotary evaporation, and the product was purified using a silica gel column with an eluent of dichloromethane/n-hexane (*v*/*v* = 2/1) to afford a yellow powder (73% yield, 0.075 g). ^1^H-NMR (500 MHz, CDCl_3_): δ 10.05 (s, 1H, -CHO), 7.96 (d, 2H, *J* = 3.5 Hz, Ar-H), 7.84 (d, 2H, *J* = 8.0 Hz, Ar-H), 7.79 (s, 1H, coumarin-H), 7.78 (d, 2H, *J* = 4.0 Hz, Ar-H), 7.69 (d, 2H, *J* = 8.5 Hz, Ar-H), 7.33 (d, 1H, *J* = 8.5 Hz, coumarin-H), 7.59 (dd, 1H, *J* = 4.0, 2.5 Hz, coumarin-H), 6.51 (d, 1H, *J* = 4.0 Hz, coumarin-H), 3.36 (t, 4H, *J* = 7.5 Hz, -NCH_2_), 1.64–1.58 (m. 4H, -CH_2_), 1.42-1.35 (m, 4H, -CH_2_), 1.00 (t, 6H, *J* = 7.5 Hz, -CH_3_); ^13^C NMR (125 MHz, CDCl_3_), δ 191.89, 161.65, 156.26, 151.14, 146.71, 140.66, 138.17, 136.14, 135.22, 130.32, 128.79, 127.54, 127.25, 121.69, 119.72, 109.19, 108.98, 97.21, 51.10, 29.29, 20.27, 13.95.

### 3.4. Synthesis of ICM

A mixture of compound **2** (0.12 g, 0.25 mmol, 1 eq), diphenylacetylene (0.07 g, 0.41 mmol, 1.64 eq), AgBF_4_ (0.10 g, 0.51 mmol, 2 eq), [RhCp*Cl_2_]_2_ (0.008 g, 0.041 mmol), 1,6-hexanediamine (0.01 g, 0.10 mmol, 0.4 eq), and Cu(OAc)_2_ (0.07 g, 0.41 mmol, 1.64 eq) was dissolved in a mixture of tert-amyl alcohol and water (5 mL, 150 µL). The reaction mixture was heated to 110 °C and stirred for 6 h. The solvent was removed via rotary evaporation, and the crude product was purified by means of column chromatography on alumina using an eluent mixture of chloroform and methanol (*v*/*v* = 3/1). After removal of the solvent via rotary evaporation, a red-brown powder (0.04 g, 11%) was obtained. The product was further dried under vacuum. ^1^H-NMR (500 MHz, CDCl_3_): δ 11.53 (s, 2H, pyridinium-H), 9.10 (d, 2H, *J* = 8.0 Hz, coumarin-H), 7.82–7.74 (m, 8H, Ar-H, coumarin-H), 7.61 (d, 4H, *J* = 8.0 Hz, Ar-H), 7.32–7.29 (m, 16H, Ar-H), 7.16 (m, 8H, Ar-H), 6.59 (d, 2H, *J* = 9.0 Hz, coumarin-H), 6.50 (s, 2H, coumarin-H), 4.78 (d, 4H, *J* = 4.5 Hz, -NCH_2_), 3.77–3.33 (m, 8H, -NCH_2_), 2.21–2.04 (m, 8H, -CH_2_), 1.61–1.58 (m, 4H, -CH_2_), 1.39–1.26 (m, 12H, -CH_2_), 0.99–0.94 (m, 12H, -CH_3_); ^13^C NMR (125 MHz, CDCl3), δ 161.52, 156.34, 151.61, 151.29, 149.33, 143.90, 140.94, 138.91, 138.11, 137.64, 137.37, 133.28, 132.92, 130.78, 130.27, 130.12, 129.12, 129.00, 128.91, 128.75, 128.57, 127.84, 126.64, 123.17, 119.26, 109.21, 109.27, 108.91, 97.18, 58.76, 51.11, 30.82, 29.28, 24.99, 20.26, 13.94; MALDI-MS calculated for C94H92N4O4 [M]^+^: 1340.719134, found: 1340.705221.

### 3.5. Measurement of Reactive Oxygen Species (ROS) Generation Capacity

#### 3.5.1. Measurement of Total ROS Generation Capacity

A 40 μM solution of 2′,7′-dichlorodihydrofluorescein (DCFH) in phosphate buffer saline (PBS) was prepared and stored on ice in the dark. ICM solution (99% H_2_O and 1% DMSO) was added to the DCFH solution to achieve a final concentration of 50 nM. The solution was transferred to disposable cuvettes and exposed to continuous white light at an intensity of 5 mW/cm^2^ for 5 min, with fluorescence measurements taken every minute at an excitation wavelength of 488 nm. The changes in fluorescence intensity were positively correlated with the ROS generation capacity, which was determined by analyzing the collected fluorescence intensity data. The blank group consisted of a 40 µM DCFH solution without any compounds, but the remaining steps were identical to those used for the experimental group.

#### 3.5.2. Measurement of Hydroxyl Radical (HO^•^) Generation Capacity

A 5 mM hydroxyphenyl fluorescein (HPF) solution was diluted with N, N-dimethylformamide (DMF) to a final concentration of 5 μM using PBS as the dilution solvent. ICM solution was added to the HPF solution to achieve a final concentration of 0.5 μM. The solution was exposed to continuous white light at an intensity of 5 mW/cm^2^ for 5 min, and the fluorescence intensity changes at 515 nm upon excitation at 490 nm were measured at intervals of 1 min. The blank group consisted of a 5 μM HPF solution without any compounds, but the remaining steps were identical to those used for the experimental group.

#### 3.5.3. Measurement of Singlet Oxygen (^1^O_2_) Generation Capacity

A 5 mM solution of Singlet Oxygen Sensor Green (SOSG) was prepared in methanol by adding 33 μL of the methanol to 100 μg of SOSG. ICM solution was added to the SOSG solution to achieve a final concentration of 0.5 μM. The solution was exposed to continuous white light at an intensity of 5 mW/cm^2^ for 5 min, and the fluorescence intensity changes at 525 nm upon excitation at 488 nm were measured at intervals of 1 min. The blank group consisted of a 5 μM SOSG solution without any compounds, but the remaining steps were identical to those for the experimental group.

#### 3.5.4. Measurement of Superoxide Anion (O_2_^•−^) Generation Capacity

ICM solution was added to a 5 μM dihydrorhodamine 123 (DHR123) DMSO solution to achieve a final concentration of 0.5 μM. The O_2_^•−^ generation capacity was determined by measuring the fluorescence intensity changes of DHR123 at 526 nm upon excitation at 495 nm. The solution was exposed to continuous white light at an intensity of 5 mW/cm^2^ for 5 min, and fluorescence intensity changes were measured at intervals of 1 min. The blank group consisted of a DHR123 solution without any compounds, but the remaining steps were identical to those for the experimental group.

#### 3.5.5. Measurement of ^1^O_2_ Quantum Yield

A total of 5 μL 9,10-anthracenediyl-bis-(methylene)dimalonic acid (ABDA) (10 mM) was added to 1 mL of ICM as a ^1^O_2_ indicator. Rose bengal (RB) was used as a standard reference. The solution was exposed to white light (5 mW/cm^2^) every minute, and the decrease in absorbance of ABDA at 378 nm was monitored. The maximum absorbance of ICM was adjusted to ~0.2 OD. The ^1^O_2_ quantum yield was calculated using the following formula:

Φ_ICM_ = Φ_RB_ × K_ICM_ × A_RB/_(K_RB_ × A_ICM_)

where K_ICM_ and K_RB_ are the decomposition rate constants of ABDA by ICM and RB, respectively, and A_ICM_ and A_RB_ are the integral area of UV–vis absorption spectra in the range of 400–700 nm. Φ_ICM_ and Φ_RB_ are the ^1^O_2_ quantum yields of ICM and RB, respectively [36,37].

### 3.6. Cultivation of Bacteria and Fungi

A single colony of bacteria/fungi from a solid medium was transferred, using an inoculating loop, into 10 mL of a liquid medium (*S. aureus*: BHI (brain heart infusion medium); *E. coli*: LB (luria-bertani medium); *C. albicans*: YPD (yeast extract peptone dextrose medium)). The culture was incubated overnight at 37 °C with continuous shaking at 180 rpm. The bacterium/fungus suspension was then centrifuged at 4500 rpm for 10 min, and the supernatant was removed. The pellet was resuspended in 1× PBS and washed twice via repeated pipetting. The bacterium/fungus cells were resuspended in 1× PBS, and the optical density at 600 nm (OD_600_) was measured. The bacterial suspension was diluted with 1× PBS to obtain a specific OD_600_ (*S. aureus*: 1.0; *E. coli*: 1.0; *C. albicans*: 2.0).

### 3.7. Photodynamic Antimicrobial Experiment

A volume of 100 μL of bacterial suspension (OD600: *S. aureus* = 1.0; *E. coli* = 1.0; *C. albicans* = 2.0) was mixed with 400 μL of 1× PBS and then treated with different concentrations of ICM. The mixture was incubated at 37 °C for 15 min. White light (5 mW/cm^2^) was applied for 15 min after the treatment with ICM. The resulting bacterial solution was subsequently diluted 10,000-fold with 1× PBS. A volume of 100 μL of the diluted bacterial solution was evenly spread on a solid culture medium. After incubation at 37 °C for 14 h, the bacterial colony count was determined. The control group was of an antibacterial experiment conducted under dark conditions, and the blank group consisted of bacterial suspension without the addition of any antibacterial agent. The procedures for the control and blank groups were identical to those for the experimental groups.

### 3.8. Zeta Potential Measurement

According to the method detailed in Section 3.7, after the interaction of *S. aureus*, *E. coli*, and *C. albicans* with different concentrations of ICM at 37 °C for 15 min, each mixture was centrifuged (7100 rpm, 10 min) to remove unbound ICM. Each sample was resuspended in 1 mL of water and sonicated repeatedly. The sample was then placed on ice for further use. For the control group, the zeta potential measurement was performed without the addition of any antimicrobial agent. The rest of the procedures for the control group were the same as those for the experimental groups.

### 3.9. Scanning Electron Microscopy (SEM) Characterization

According to the method detailed in Section 3.7, after the interactions of *S. aureus*, *E. coli*, and *C. albicans* with different concentrations of ICM, each mixture was centrifuged (7100 rpm, 10 min) to remove the supernatant, and the pellet was resuspended in 100 µL of water. A total of 2 μL sample was dropped onto a silicon chip, which was then left to air-dry on a super-clean bench. The sample was then fixed overnight with a 0.1% glutaraldehyde solution. After two rinses with pure water (each time for 6 min), the sample was dehydrated sequentially with 40%, 70%, 90%, and 100% ethanol (6 min each). After air-drying at room temperature and freeze-drying for 2 h, the silicon chip was affixed to a sample holder using conductive adhesive and sprayed with gold before analysis.

### 3.10. Confocal Laser Scanning Microscopy (CLSM) Imaging

According to the method detailed in Section 3.7, each sample was resuspended in 10 µL 1× PBS, and 5 µL of the solution was dropped onto a glass slide. A cover slide was applied, and the sample was observed using a fluorescence microscope. The objective magnification was set to 100×. ICM’s excitation wavelength is 488 nm, and the emission of ICM was collected from 550 nm to 650 nm using blue as a false color.

### 3.11. Biocompatibility

NIH-3T3 cells (mouse embryonic fibroblast cells) were seeded at a density of 5000 cells per well in a 96-well plate. After incubation in a 5% CO_2_ incubator for 24 h, the treatment was administered and the cells were incubated for a further 24 h. Afterward, a CCK-8 (cell counting kit-8) reagent was added, and the plate was incubated in the dark for 2 h. The absorbance values (OD_450_) were then measured.

## 4. Conclusions

In summary, we developed an AIEgen, named ICM, that exhibits highly efficient generation of type I ROS (HO^•^ and O_2_^•−^) for antibacterial therapy, effectively killing *S. aureus* under a low light intensity. Structurally, ICM comprises a dual D-π-A architecture connected by alkyl chains, reducing the energy gap to facilitate ISC. Additionally, the incorporation of two cationic groups enables binding to pathogenic microorganisms via electrostatic interactions. The fluorescence imaging, zeta potential, and SEM results collectively indicate that ICM interacts with bacterial cell membranes through electrostatic interactions. Furthermore, the significant generation of type I/II ROS generation by ICM allows for the complete eradication of *S. aureus* at a low light intensity (5 mW/cm^2^) with an ICM concentration of 2 µM. Therefore, ICM displays potential as an effective treatment for pathogenic microorganisms under hypoxic conditions in vivo, and our methods provide a feasible strategy for designing efficient type I/II photosensitizers.

## Data Availability

The original contributions presented in the study are included in the article (and Supplementary Material), further inquiries can be directed to the corresponding authors.

## Supplementary Materials

The following supporting information can be downloaded at: https://www.mdpi.com/article/10.3390/molecules29163793/s1. Figure S1: ^1^H NMR spectrum of compound 1 in CDCl_3_; Figure S2: ^13^C NMR spectrum of compound 1 in CDCl_3_; Figure S3: ^1^H NMR spectrum of compound 2 in CDCl_3_; Figure S4: ^13^C NMR spectrum of compound 2 in CDCl_3_; Figure S5: ^1^H NMR spectrum of ICM in CDCl_3_; Figure S6: ^13^C NMR spectrum of ICM in CDCl_3_; Figure S7: HRMS spectrum of ICM; Figure S8: Normalized UV–vis spectra in different solvents; Table S1: The optical of ICM; Figure S9: The UV-vis absorption spectra of (A) ICM and (B) RB with ABDA at various irradiation time; integral areas of (C) ICM and (D) RB; corresponding linear fit-curves for (E) ICM and (F) RB under white light irradiation (5 mW/cm^2^); Figure S10: (A) *C. albicans* and different concentrations of ICM on agar plates under light (5 mW/cm^2^) and dark conditions; (B) *E. coli* and different concentrations of ICM on agar plates under light (5 mW/cm^2^) and dark conditions; (C) survival rate graph of *C. albicans* treated with different concentrations of ICM; (D) survival rate graph of *E. coli* treated with different concentrations of ICM; (E) zeta potential of *C. albicans* treated with different concentrations of ICM; (F) zeta potential of *E. coli* treated with different concentrations of ICM; (G) SEM images of ICM interacting with different pathogenic microorganisms (scale bar: 1 μm); Figure S11: Viability rates of NIH-3T3 cells treated with different concentrations of compounds.
