# Supplementary material for: Synthesis of Coumarin-Based Photosensitizers for Enhanced Antibacterial Type I/II Photodynamic Therapy
Source: Molecules. 2024 Aug 10;29(16):3793. doi: 10.3390/molecules29163793 (PMC11357021; doi:10.3390/molecules29163793)
Supplement: Supplementary file 1 [file molecules-29-03793-s001.zip › molecules-3082028-supplementary.pdf]

Supporting Information Synthesis of Coumarin-based Photosensitizers for Enhanced Antibacterial Type Ⅰ/Ⅱ Photodynamic Therapy

Min Ma [1], Lili Luo [1], Libing Liu [1,2,*], Yuxuan Ding [1], Yixuan Dong [1] and Bing Fang [1,2,*]

1 Department of Nutrition and Health, China Agricultural University, Beijing 100193, China; mamin_st@163.com (M.M.); lililuo_w@163.com (L.L.); dingyxuan2024@163.com (Y.D.); dongyuxuan322@163.com (Y.D.)
2 Key Laboratory of Precision Nutrition and Food Quality, China Agricultural University, Beijing 100193, China
* Correspondence: liulibing@cau.edu.cn (L.L.); bingfang@cau.edu.cn (B.F.)

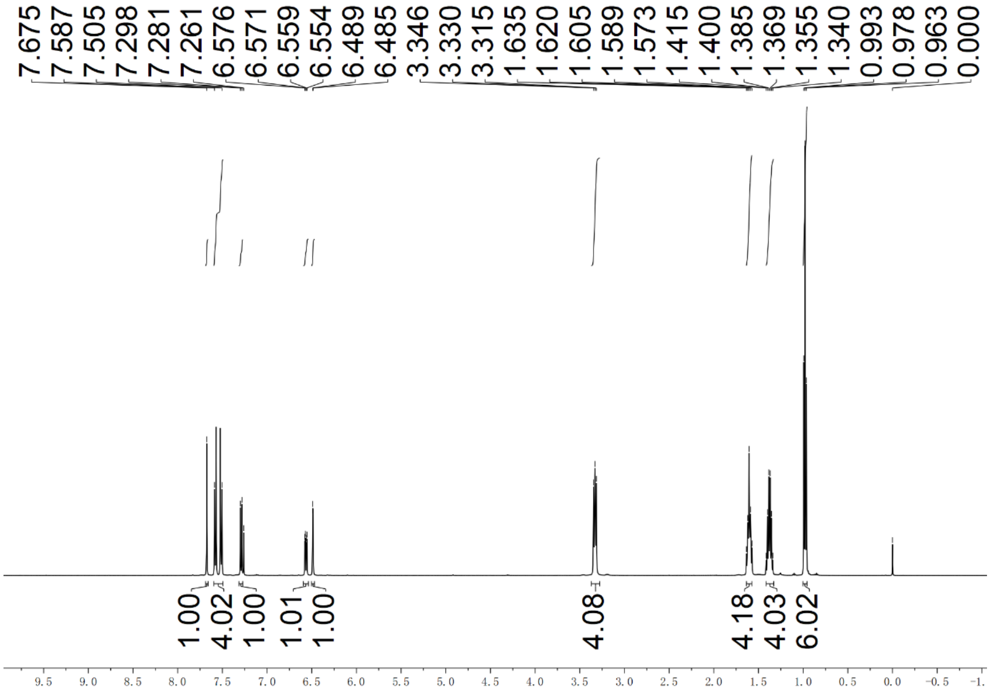

Figure S1. ^{1}H NMR spectrum of compound 1 in $CDCl_3$.

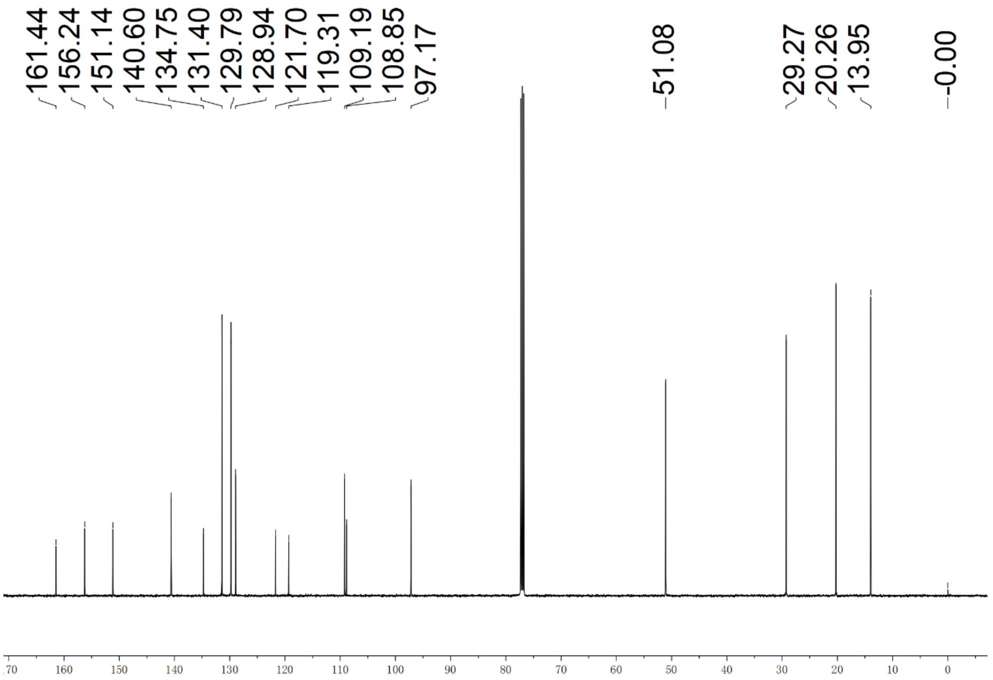

Figure S2. ^{13}C NMR spectrum of compound 1 in $CDCl_3$.

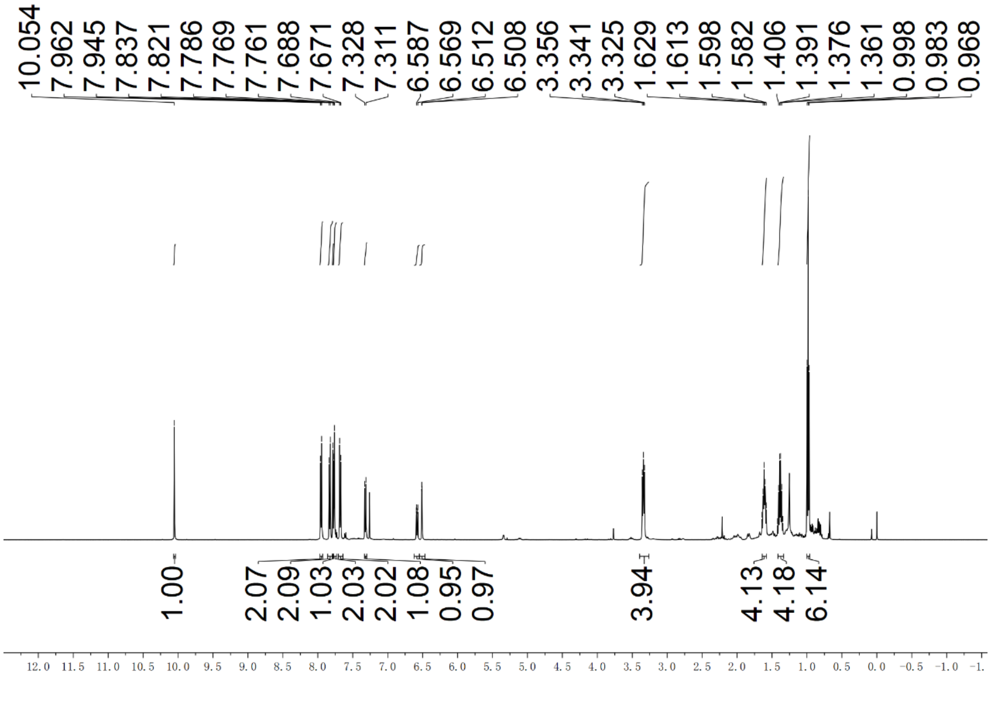

Figure S3. ^{1}H NMR spectrum of compound 2 in $CDCl_3$.

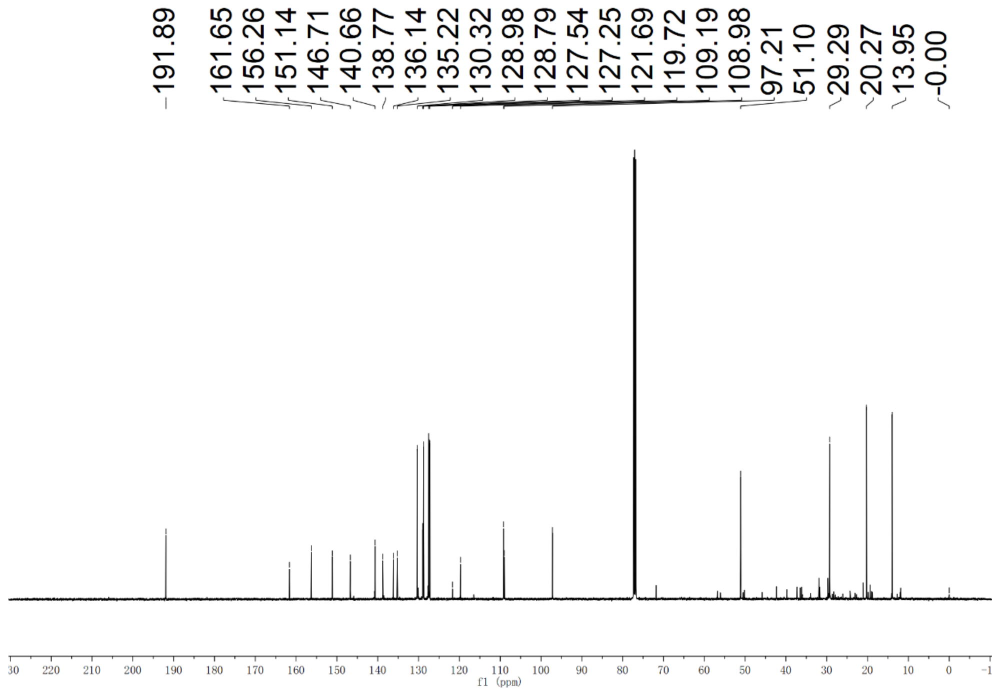

Figure S4. ^{13}C NMR spectrum of compound 2 in $CDCl_3$.

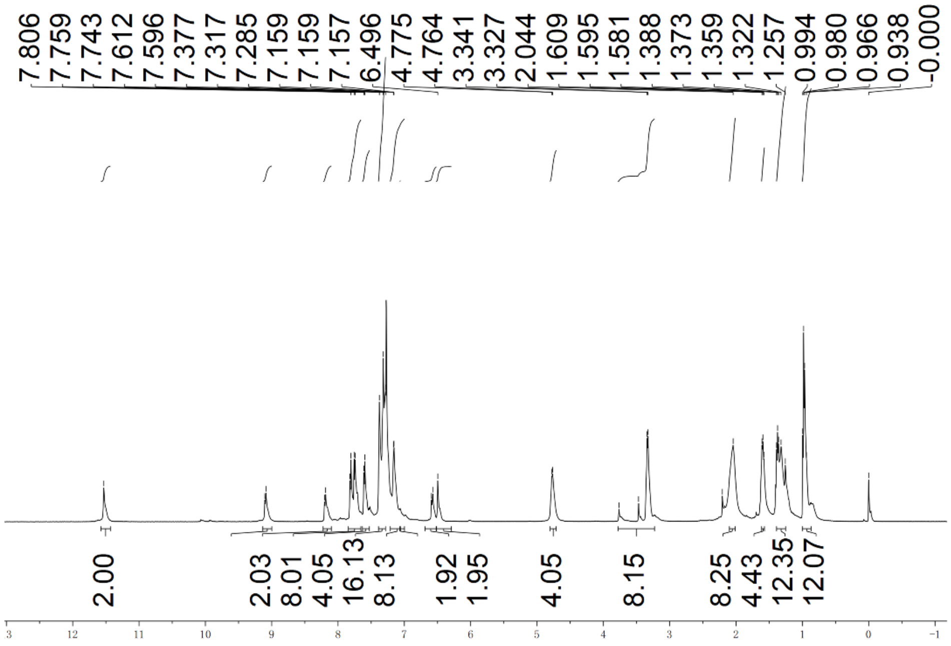

Figure S5. ^{1}H NMR spectrum of ICM in $CDCl_3$.

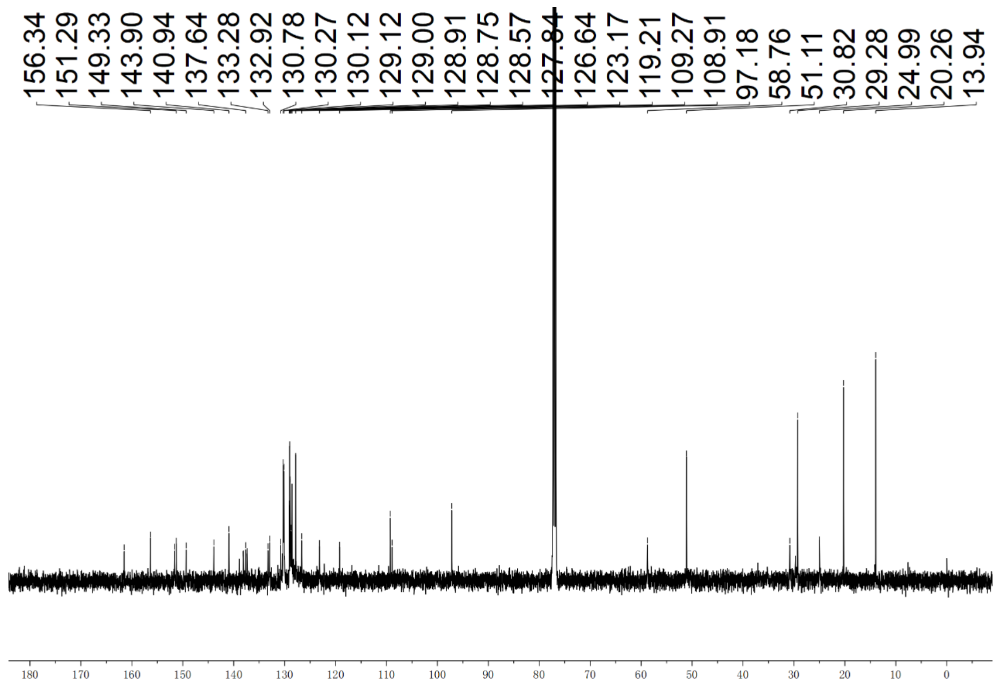

Figure S6. ^{13}C NMR spectrum of ICM in $CDCl_3$.

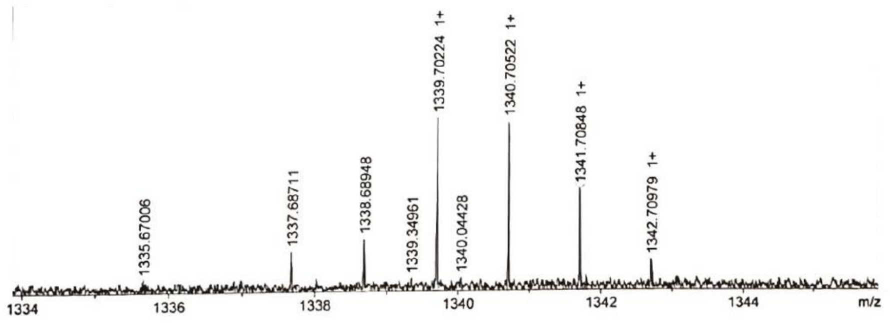

Figure S7. HRMS spectrum of ICM.

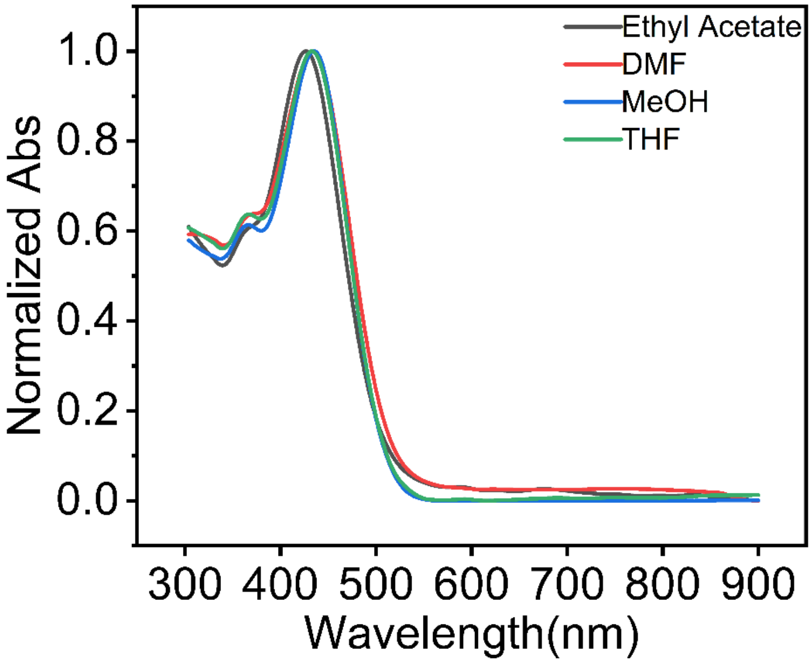

Figure S8. Normalized UV–vis spectra in different solvents.

Table S1. The optical of ICM.

Dye	Absorption (nm)	Emission (nm)	Stokes shift (nm)	Fluorescence quantum yield (%)	Molar absorption coefficient (L mol^{-1} cm^{-1})
ICM	λ^{a1}, λ^{a2}	λ^{b1}, λ^{b2}	E_{exp}^{c1}, E_{exp}^{c2}	Φ_f^{d1}, Φ_f^{d2}	ε^{e1}, ε^{e2}
	447, 418	661, 501	~214, ~83	0.102, 0.071	22000, 29800

[a] Absorption maximum; [b] emission maximum; [c] stokes shift; [d] fluorescence quantum yield; [e] molar absorption coefficient of ICM in H_2O (1)/DMSO (2).

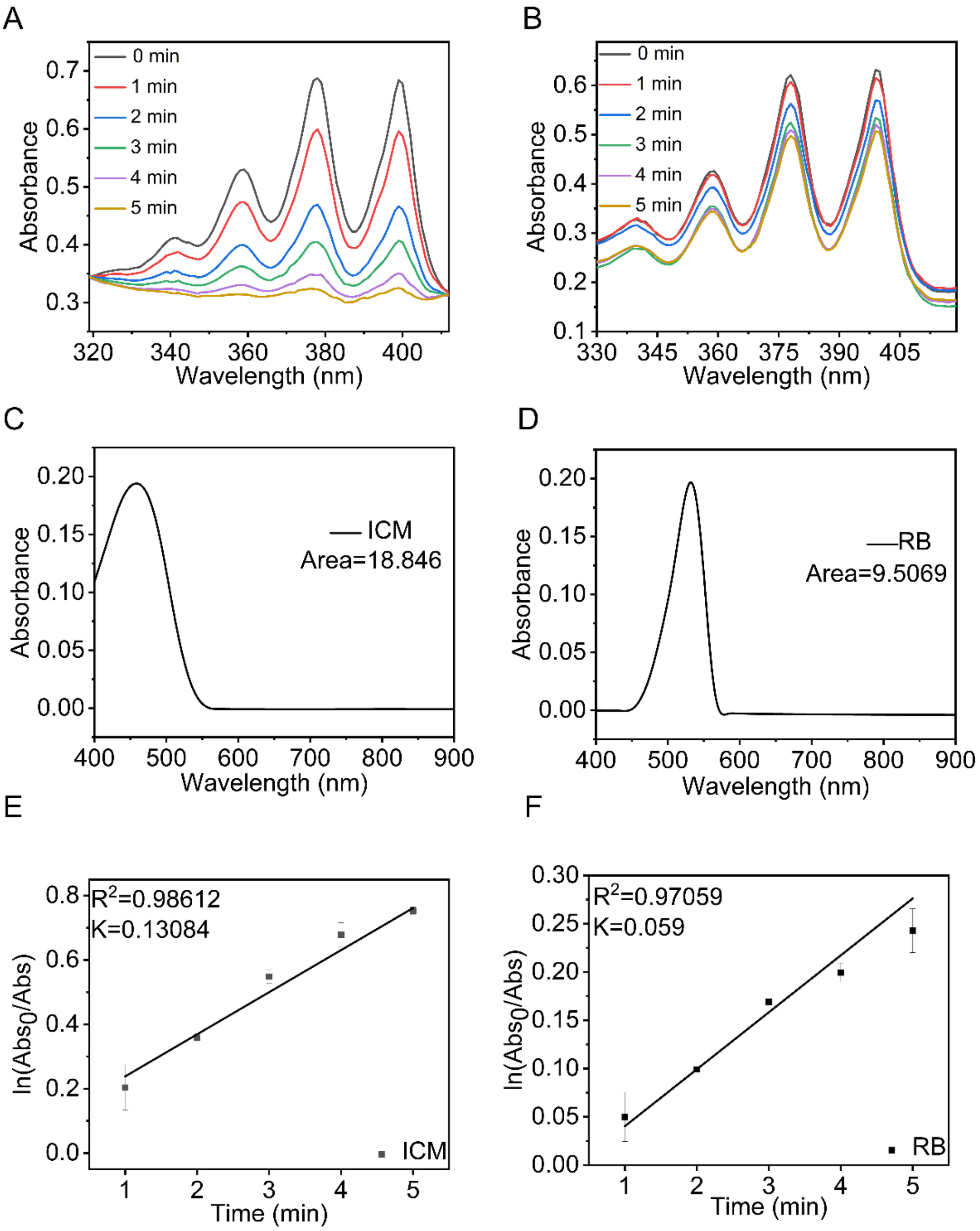

Figure S9. The UV-vis absorption spectra of (**A**) ICM and (**B**) RB with ABDA at various irradiation time; integral areas of (**C**) ICM and (**D**) RB; corresponding linear fit-curves for (**E**) ICM and (**F**) RB under white light irradiation (5 mW/cm^2).

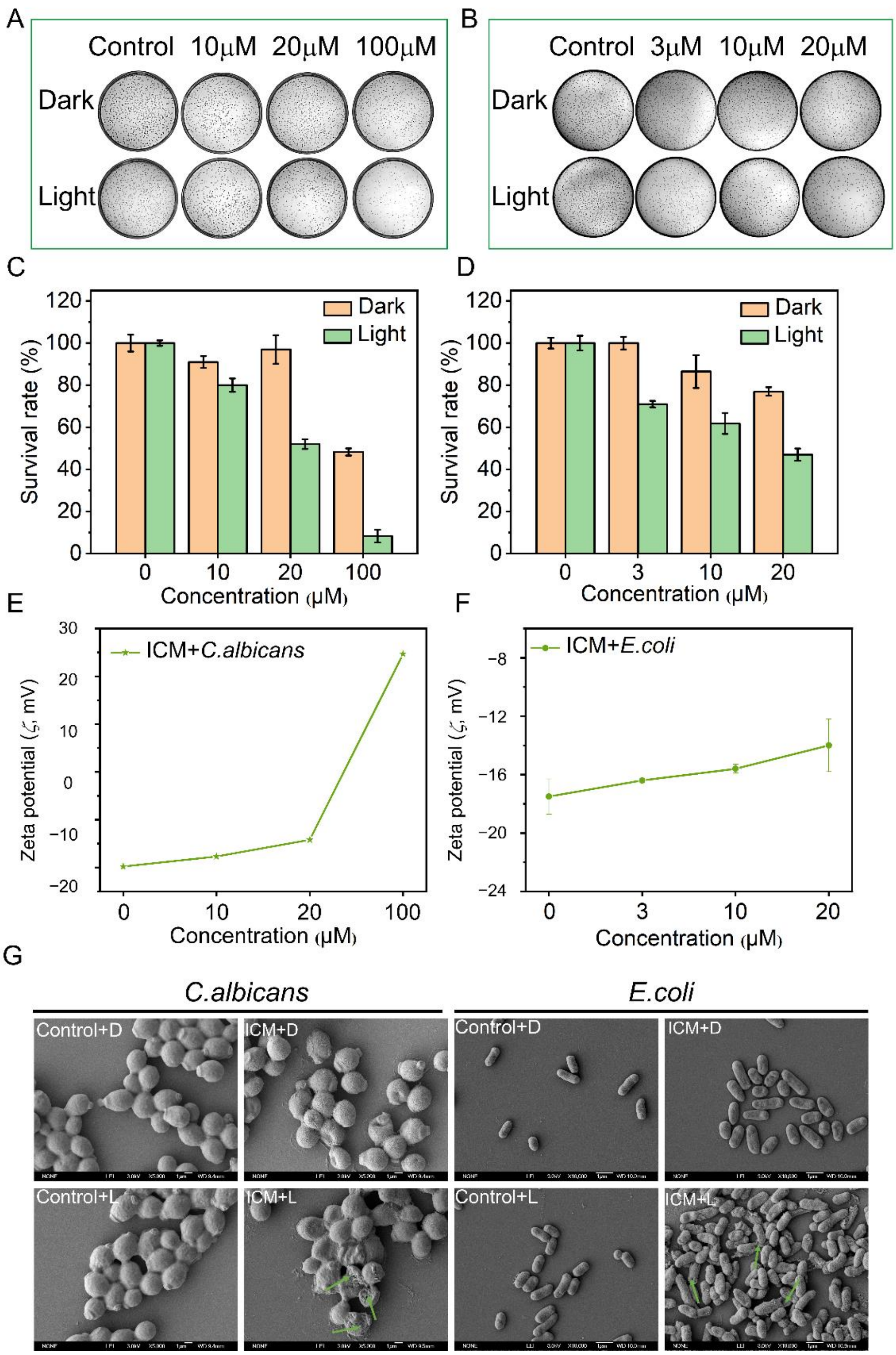

Figure S10. (**A**) *C. albicans* and different concentrations of ICM on agar plates under light (5 mW/cm^2) and dark conditions; (**B**) *E. coli* and different concentrations of ICM on agar plates under light (5 mW/cm^2) and dark conditions; (**C**) survival rate graph of *C. albicans* treated with different concentrations of ICM; (**D**) survival rate graph of *E. coli* treated with different concentrations of ICM; (**E**) zeta potential of *C. albicans* treated with different concentrations of ICM; (**F**) zeta potential of *E. coli* treated with different concentrations of ICM; (**G**) SEM images of ICM interacting with different pathogenic microorganisms (scale bar: 1 μm).

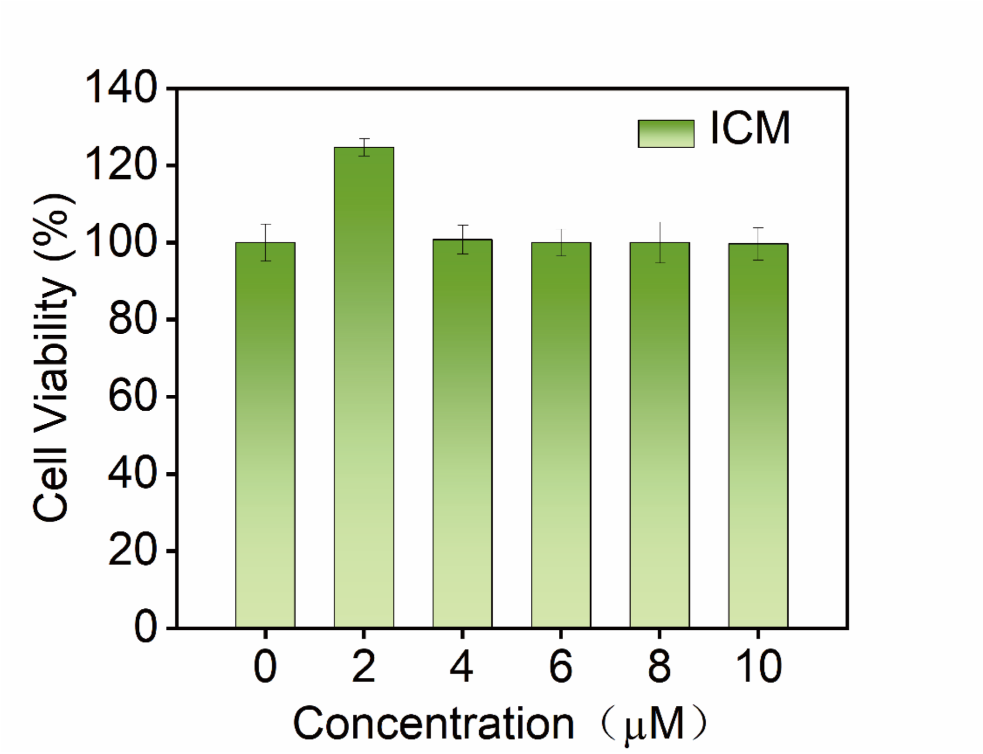

Figure S11. Viability rates of NIH-3T3 cells treated with different concentrations of compounds.
